# Computerized alcohol screening identified more at-risk drinkers in a level 2 than a level 1 trauma center

**DOI:** 10.1186/s12889-016-3989-6

**Published:** 2017-01-06

**Authors:** Ghasem Imani, Cristobal Barrios, Craig L. Anderson, Maryam Hosseini Farahabadi, Faried Banimahd, Bharath Chakravarthy, Wirachin Hoonpongsimanont, Christopher E. McCoy, Georginne Mercado, Babak Farivar, Jacqueline K. Pham, Shahram Lotfipour

**Affiliations:** 1 Department of Emergency Medicine, University of California Irvine, Orange, CA USA; 2 The Division of Trauma, Burns, Critical Care and Acute Care Surgery, University of California Irvine, Orange, CA USA; 3 Trauma Services at St. Joseph Health, Mission Viejo, CA USA

**Keywords:** Level 1 trauma center, Level 2 trauma center, Computerized alcohol screening, At-risk drinking

## Abstract

**Background:**

Alcohol abuse is recognized as a significant contributor to injury. It is therefore essential that trauma centers implement screening and brief intervention (SBI) to identify patients who are problem drinkers. Although, the utility of SBI in identifying at-risk drinkers have been widely studied in level 1 trauma centers, few studies have been done in level 2 centers. This study evaluates the usefulness of SBI in identifying at-risk drinkers and to investigate the pattern of alcohol drinking among level 2 trauma patients.

**Methods:**

This is a retrospective study of a convenience sample of trauma patients participating in computerized alcohol screening, brief intervention, and referral to treatment (CASI) in an academic level 1 trauma center and a nearby suburban community hospital level 2 trauma center. CASI utilized Alcohol Use Disorders Identification Test (AUDIT) to screen patients. We compared the pattern of alcohol drinking, demographic factors, and readiness-to-change scores between those screened in a level 2 and 1 trauma center.

**Results:**

A total of 3,850 and 1,933 admitted trauma patients were screened in level 1 and 2 trauma centers respectively. There was no difference in mean age, gender, and language between the two centers. Of those screened, 10.2% of the level 1 and 14.4% of the level 2 trauma patients scored at-risk (AUDIT 8–19) (*p* < 0.005). Overall, 3.7% of the level 1 and 7.2% of the level 2 trauma patients had an AUDIT score consistent with dependency (AUDIT > =20) (*p* < 0.005). After adjusting for age, sex, education, and language, the odds of being a drinker at the level 2 center was two times of those at the level 1 center (*p* < 0.005). The odds of being an at-risk or dependent drinker at level 2 trauma center were 1.72 times of those at the level 1 center (*p* < 0.005).

**Conclusions:**

Findings suggest that SBI is effective in identifying at-risk drinkers in level 2 trauma center. SBI was able to identify all drinkers, including at-risk and dependent drinkers at higher rates in level 2 versus level 1 trauma centers. Further studies to evaluate the effectiveness of SBI in altering drinking patterns among level 2 trauma patients are warranted.

## Background

Excessive alcohol consumption is a risk factor for many health and societal problems. It accounts for the third leading cause of death in the United States, which is in fact preventable [1]. According to the Centers for Disease Control and Prevention (CDC), almost 30 people die each day in motor vehicle crashes that involve an alcohol-impaired driver in the United States. This is almost one death every 51 min [2]. In 2013, 10,076 people were killed in alcohol-related driving accidents, which accounts for 31% of all annual traffic-related deaths in the US [3]. Almost $59 billion is spent annually on alcohol-related crashes [4].

According to the CDC, the Community Preventive Services Task Force has suggested multiple programs to reduce harmful alcohol use [5]. These measures include, the regulation of alcohol outlet density, increasing alcohol taxes, dram shop liability, maintaining limits on days of sale, maintaining limits on hours of sale, enhanced enforcement of laws prohibiting sales to minors, and electronic screening and brief intervention (e-SBI) [5]. Screening and behavioral counseling intervention is recommended by the United States Preventive Services Task Force (USPSTF) to be done in primary care in order to target individuals whose drinking patterns does not fall into alcohol dependence range but place them at risk of alcohol-related harms [6].

According to the previous data, a quarter of the US population is at-risk drinker [7].

Additionally, the prevalence of alcohol, tobacco, and other drug (ATOD) use problems among emergency department (ED) patients is 50–100% higher than the U.S. average [8, 9]. A large percentage of ED patients have unrecognized ATOD evaluation needs and are more likely to be frequently admitted to the hospital or repeatedly utilize ED services [9, 10]. Prior research has shown that at least 25% of all adult ED patients screen positive for hazardous or harmful drinking pattern [11, 12]. Alcohol use problems are endemic among injured trauma patients as well [13, 14]. Thus, the widespread integration of alcohol screening and brief intervention in ED and trauma centers not only can identify those at risk, but could also be a powerful preventive tool, which has a long-standing public health impact [15, 16].

Multiple instruments are widely used to screen for alcohol drinking problems such as the Alcohol Use Disorders Identification test (AUDIT), AUDIT-C [17], and the Alcohol Smoking and Substance Involvement Screening Test (ASSIST) [18]. Each of these questionnaires can be utilized as a computerized version. The computerized alcohol screening, brief intervention, and referral to treatment (CASI) has been introduced by the University of California, Irvine in 2006 and has been used to screen at-risk drinkers at the ED since then. The utility of this program in screening at-risk drinkers has been widely studied in different settings [19–22].

Based on the National Trauma Data Bank 2015, there are 237 level 1 and 259 level 2 trauma centers in the US where the trauma patients receive care [23]. Alcohol SBI has been mandated by the American College of Surgeons to be done in all level 1 trauma centers since 2006 [24]. While many of these trauma patients may have alcohol and drug problems, SBI for alcohol is not mandatory in level 2 trauma centers, but only recommended. As a result, most of the studies have evaluated the effectiveness of SBI done in level 1 trauma centers while very little documentation exists regarding its use at level 2 trauma centers [25]. That means a great proportion of trauma patients who are seen at level 2 trauma centers are not offered SBI.

In this study, we hypothesize that in a level 2 community trauma center, SBI will perform its task of alcohol use disorder detection consistent with results seen at level 1 trauma centers. For this purpose, we compared the pattern of alcohol drinking, demographic factors and readiness-to-change scores between those screened in a level 2 and a level 1 trauma center using computerized SBI. If it proves to be effective in identifying alcohol use disorder in level 2 trauma centers, further studies to evaluate the usefulness of brief intervention in altering drinking patterns among these patients would be warranted and screening and brief intervention (SBI) could become standard of care in all level 2 trauma centers as well.

## Methods

### Study design, study setting and population

This is a retrospective study of a convenience sample of trauma patients participating in computerized SBI in a level 1 comparing to a level 2 trauma center in Orange County, California. The University of California, Irvine Human Subjects Research Institutional Review Board reviewed and approved the study protocol and waived the need for consent (HS#2014-1330). We collected data from a level 1 university hospital and a nearby suburban community hospital level 2 Trauma center in Southern California from 2010 to 2014 inclusive.

While the trained research personnel implemented computerized SBI using a touchscreen tablet computer on all designated trauma patients admitted to the hospital in the level 1 trauma center, nurses were responsible to perform this screening in the level 2 trauma center. Patients were screened seven days a week, without interfering with patient care. All adult (age 18 and above) designated trauma patients were eligible for SBI. The computerized SBI module consists of a bilingual (English and Spanish) audio-graphical interface software program that was up-loaded onto a mobile tablet computer and administered at the bedside of stable trauma patients in the inpatient trauma units. It uses dynamic text, touch screen technology, and offers a text-to speech option. Headphones with Bluetooth technology are also available for patient privacy. It also recorded patients’ responses and the length of time with the module. A personalized alcohol-reduction plan, along with counseling referral information, was wirelessly printed on a department printer [20, 21].

The computerized interview began with the first three questions of the Alcohol Use Disorders Identification Test (AUDIT) [26] plus an additional question about the highest number of drinks in the last month. The possible responses included the AUDIT categories for less frequent drinking, but the program inquired about the exact number of drinking days per week and the number of drinks per drinking day. The responses were used to calculate drinks per week and to assess the points for the first two AUDIT questions. The computerized SBI determined whether the patient drank more than the National Institute on Alcohol Abuse and Alcoholism (NIAAA) recommended thresholds, defined as no more than four drinks in a day and no more than fourteen drinks in a week for men age 64 years and younger and no more than three drinks in a day and no more than seven drinks in a week for women and for men age 65 years and older [27]. Patients who reported exceeding NIAAA limits were presented with the remaining AUDIT questions and the results of the screening were included in this study. Then, they are asked about their readiness-to-change their alcohol drinking habit.

### Variables

Trauma centers: According to the American Trauma Society, “A Level 1 Trauma Center is capable of providing total care for every aspect of injury – from prevention through rehabilitation. Elements of Level 1 Trauma Centers Include: 24-h in-house coverage by general surgeons, and prompt availability of care in specialties such as orthopedic surgery, neurosurgery, anesthesiology, emergency medicine, radiology, internal medicine, plastic surgery, oral and maxillofacial, pediatric and critical care” [28].

“A Level 2 Trauma Center is able to initiate definitive care for all injured patients.

Elements of Level 2 Trauma Centers Include: 24-h immediate coverage by general surgeons, as well as coverage by the specialties of orthopedic surgery, neurosurgery, anesthesiology, emergency medicine, radiology and critical care, tertiary care needs such as cardiac surgery, hemodialysis and micro vascular surgery may be referred to a Level 1 Trauma Center [28].

Demographic data: Age is defined in years. It is also categorized into 6 different groups (18-20, 21-29, 30-39, 40-49, 50-64, 65+). Sex is defined as male or female. Language is reported in two categories: English and Spanish. This is the preferred language in which patients took the test. Education was categorized into four groups: Less than high school, high school graduate, some college/associate degree, and 4 years/advanced degree. “Some college/associate degree” serves as our reference group.

AUDIT score: This score ranges from 0-40. A score of 1-7 is categorized as low-risk although all of these patients in this sample exceeded the NIAAA recommendation levels. Scores 8-19 identifies individuals who are considered as potentially at risk for alcohol-related injuries and side effects. Pattern of alcohol dependency is defined with scores 20 and higher [26].

Readiness- to-change score (RTC): This score is a patient self-reported score using a 1-10 Likert scale [29]. Patients would be asked for their stage of change only if they report drinking above the NIAAA drinking limit. We then categorized RTC into three levels of readiness to change, minimal (scores 1 to 3), moderate (scores 4 to 7) and high (scores 8 to 10), corresponding to peaks in RTC at 1, 5, and 10 [29].

### Data analysis

AUDIT Screening results and RTC scores as well as age, sex, education, language of interview were recorded by the SBI program in a comma-separated file, imported into Stata (version 14.0, StataCorp, College Station, TX), compared and then analyzed. The values were grouped into categories, which allowed the analysis to identify and present nonlinear trends without complex nonlinear models. We used the chi-square test for independence to compare demographic variables between the two trauma centers. Logistic regression was used for multivariate modeling of these associations.

## Results

### Patient enrollment

Overall, 4,282 and 1,998 admitted trauma patients at the level 1 and 2 trauma centers were screened using the computerized SBI during the day until midnight. We were not able to approach all eligible trauma patients due to the unavailability of research personnel over night. Among these patients, 38 (0.9%) patients at the level 1 and 11 (0.5%) at the level 2 refused to participate in the study. Among those agreed to participate, 394 (9.2%) patients at the level1 and 54 (2.7%) at the level 2 trauma centers did not complete the survey. A total of 5,783 admitted trauma patients from 2010 through 2014 were enrolled in the study. Among these, 3,850 were enrolled in the level 1 and 1,933 in the level 2 trauma center.

### Demographics

Patients’ demographic characteristics are shown in the Table 1. The level 2 trauma center had more patients age 40–49 and 50–64 than the level 1 center. We observed approximately twice as many males as females in both level 1 and 2 trauma centers respectively. A total of 94.6% of patients at the level 1 and 94.5% of those at the level 2 trauma centers answered the education question. Results showed that patients at the level 2 trauma center reported higher education level; more had some college or an associate, 4-years, or advanced degree. Nearly 94% of those screened at the both trauma centers chose to take the test in English, the rest of the patients took it in Spanish.

**Table 1:** Baseline demographic characteristics

Variables (N total = 5783)	N/Total (%)	Level 1 trauma center (%)	Level 2 trauma center (%)	*P*-value
Age				
18–20	517(8.9)	346(9.0)	171(8.8)	0.001
21–29	1279(22.1)	846(22.0)	433(22.4)	
30–39	755(13.1)	530(13.8)	225(11.6)	
40–49	848(14.7)	529(13.7)	319(16.5)	
50–64	1225(21.2)	787(20.4)	438(22.7)	
65+	1159(20.0)	812(21.1)	347(18.0)	
Sex				
Male	3913(67.7)	2602(67.6)	1311(67.8)	0.85
Female	1870(32.3)	1248(32.4)	622(32.2)	
Education				
Less than high school	511(8.8)	392(10.2)	119(6.2)	<.001
High school graduate	1876(32.5)	1330(34.6)	546(28.2)	
Some college/Associate degree	1835(31.7)	1200(31.2)	635(32.8)	
4 year/advanced degree	1318(22.8)	791(20.4)	527(27.3)	
Language				
Spanish	362(6.3)	244(6.3)	118(6.1)	0.73
English	5421(93.7)	3606(93.7)	1815(93.9)	
Drinking level				
Non drinker	1955(33.8)	1489(38.7)	466(24.1)	<.001
Within NIAAA limit	2207(38.2)	1375(35.7)	832(43.0)	
Above NIAAA limit and:				
Low risk (AUDIT 1–7)	668(11.5)	450(11.7)	218(11.3)	
At-risk (AUDIT 8–19)	672(11.6)	394(10.2)	278(14.4)	
Dependent (AUDIT 20–40)	281(4.9)	142(3.7)	139(7.2)	
Readiness-to-change score				
1–3	257(17.0)	153(16.4)	104(18.0)	0.021
4–7	520(34.5)	346(37.2)	174(30.2)	
8–10	731(48.5)	432(46.4)	299(51.8)	

We found a higher non-drinkers rate among level 1 trauma center patients (38.7%) than level 2 (24.1%), more drinkers who exceed the NIAAA thresholds in the level 2 center (25.6% in level 1 and 32.9% in level 2), and more at-risk and dependent drinkers among level 2 center patients. More level 1 trauma center patients identified themselves to be moderately ready to change their drinking habits; however, more level 2 patients were strongly ready to change. The same differences in readiness to change were present when logistic regression was used to control for the other variables in Table 1.

### Demographic factors associated with being a drinker

Table 2 is an adjusted regression model showing the associated factors with being a drinker. After adjusting for sex, age, language, and education level, the odds of being a drinker of any amount in level 2 trauma center were two times those at the level 1 (odds ratio: 2.00, 95%CI 1.75–2.29).

**Table 2:** Adjusted model for association between demographic factors and being a drinker (any amount)

Drinker	Odds ratio	*P* value	95% CI	
Trauma center				
Level 1^a^	Ref	Ref	Ref	
Level 2	2.00	<0.001	1.75	2.29
Age				
18–20	0.48	<0.001	0.38	0.61
21–29^a^	Ref	Ref	Ref	
30–39	0.82	0.091	0.65	1.03
40–49	0.55	<0.001	0.44	0.68
50–64	0.34	<0.001	0.28	0.41
65+	0.18	<0.001	0.15	0.22
Sex				
Male^a^	Ref	Ref	Ref	
Female	0.50	<0.001	0.44	0.57
Education level				
Less than high school	0.80	0.055	0.64	1.00
High school graduate^a^	Ref	Ref	Ref	
Some college/Associate degree	1.32	<0.001	1.13	1.53
4 year/advanced degree	1.30	0.002	1.10	1.54
Language				
English^a^	Ref	Ref	Ref	
Spanish	0.79	0.073	0.61	1.02
Constant	4.04	<0.001	3.40	4.80

^a^Reference group for comparison (pseudo R^2^ = 0.11)

### Demographic factors associated with being at-risk or dependent drinker

As shown in the Table 3, after controlling for age, sex, language, and education level, the odds of being an at-risk or dependent drinker (AUDIT > =8) at level 2 were 1.72 times those at the level 1 trauma center (odds ratio 1.72, 95%C1 1.48–2.00).

**Table 3:** Adjusted model for association between demographic factors and showing the patterns of at-risk drinking or dependency (AUDIT > =8)

At-risk drinking or dependency	Odds ratio	*P* value	95% CI	
Trauma center				
Level 1^a^	Ref	Ref	Ref	
Level 2	1.72	<0.001	1.48	2.007
Age				
18–20	0.63	0.001	0.48	0.82
21–29^a^	Ref	Ref	Ref	
30–39	0.93	0.58	0.74	1.17
40–49	0.70	0.003	0.56	0.88
50–64	0.53	<0.001	0.42	0.66
65+	0.23	<0.001	0.17	0.31
Sex				
Male^a^	Ref	Ref	Ref	
Female	0.40	<0.001	0.33	0.49
Education level				
Less than high school	0.91	0.519	0.69	1.20
High school graduate^a^	Ref	Ref	Ref	
Some college/Associate degree	0.72	<0.001	0.60	0.86
4 year/advanced degree	0.62	<0.001	0.49	0.77
Language				
English^a^	Ref	Ref	Ref	
Spanish	0.79	0.16	0.58	1.09
Constant	0.39	<0.001	0.32	0.46

^a^Reference group for comparison (pseudo R^2^ = 0.07)

## Discussion

To our knowledge, this is one of the few studies done at a level 2 trauma center to examine the utility of SBI and to evaluate its usefulness in finding at-risk drinkers among the trauma patients seen at a level 2 trauma center [25]. Our findings suggest that SBI is successful in identifying at-risk and dependent alcohol drinkers in a level 2 trauma center. While the results showed that almost 13.9% of individuals screened in the level 1 trauma center were identified by AUDIT as at-risk or dependent, this number was 21.6% in the level 2 trauma center. The results also showed that there are more drinkers among level 2 trauma patients in all age groups compared to the level 1. It should not be overlooked that the population screened at the level 2 trauma center had higher education level. The effect of education on alcohol drinking habits is controversial. While some studies proved no association between level of education and alcohol drinking [30, 31], others showed that lower education level is associated with higher level of drinking [32]. Overall, the effect of demographic factors was not strong enough to explain the identification of higher number of drinkers found at the level 2 in our study. The odds of being a drinker of any amount is still 2.00 times in patients screened in the level 2 than the level 1 trauma center. Even assuming the same demographic factors, the likelihood of individuals being an at-risk or dependent drinker is 1.72 times in level 2 compared to those screened in level 1. Additionally, our data showed that a greater number of patients in level 2 trauma center exhibited strong willingness to change their alcohol drinking habits compared to the level 1.

For every individual in the US who is alcohol dependent, there are six other adults who are at risk for injury due to their alcohol drinking habits. As a matter of fact, this population is more likely to utilize trauma center services [33]. It has been reported that 30%–50% of trauma center cases are alcohol related [34]. SBI in primary care can result in a 15–30% reduction in alcohol consumption lasting for at least a year [35, 36]. Screening and brief intervention provided in EDs is more effective at a lower cost compared to screening provided to the outpatients [37] and proved to be effective in reducing hazardous alcohol drinking behavior at 6 months follow-up in non-dependent group screened at level 1 trauma and ED [38]. The reasons for screening level 1 trauma patients apply equally to level 2 trauma patients. In the two centers we examined, levels of alcohol use were greater at the level 2 trauma center with a greater number of patients demonstrating high level of readiness-to-change. Given the ease of computerized screening, failure to screen level 2 patients is a missed opportunity to reduce subsequent injuries among trauma patients.

Nonetheless, studies have shown that ED directors might not be familiar with the SBI or might be unaware of it being mandatory [35]. There are barriers such as time constraint and financial resources that causes the SBI to lag behind the national guidelines [39]. Alcohol related problems are the health issues that are mostly addressed by computerized health interventions [40]. Computerized screening performs better in finding at-risk drinkers in all age groups compared to medical screening examination [22]. This may be due to patient’s’ preference to disclose their sensitive health information to computers compared to health care staff and they are more likely to share honest answers [41]. It further improves patients’ knowledge of safe drinking and delivers alcohol education in a comfortable way [42].

Our study proved that SBI is as useful in finding at-risk and dependent drinkers at a level 2 trauma center as a level 1 trauma center. Therefore, we suggest further studies to evaluate the barriers to the mandatory implementation of SBI in all level 2 trauma centers.

### Limitation

This study uses convenience samples at only two institutions. Patients were not enrolled overnight or when the emergency department and trauma service experienced heavy patient volumes. The fact that we could not control for other socio economic status such as income, employment, ethnicity etc. limited our ability to analyze the differences between patients of the two centers. The focus of our study was on the feasibility of SBI in level 2 trauma center and to evaluate its usefulness in identifying at-risk drinkers. Thus, the next step in studying SBI would be to investigate the effectiveness of intervention in altering alcohol drinking behavior.

The personnel conducting the computerized model of SBI were different at the two institutions. Although the differences between institutions prevent a direct comparison, nurses may be more effective in enrolling patients and encouraging completion. This is likely due to their familiarity with the patient or being able to time the intervention to the patient’s openness to it. This may account for some of the differences we found between the two institutions.

## Conclusions

We identified that SBI performed in level 2 trauma centers was as effective in level 1 trauma centers in revealing at-risk drinkers. This population of patients was recognized as being in a vulnerable state due to the cause and event bringing them to a trauma center. Continuing to target this population with SBI and provide outreach and awareness is markedly essential in both level 1 and 2 trauma centers.

## Abbreviations

ASSIST: Alcohol smoking and substance involvement screening test
ATOD: Alcohol, tobacco, and other drug
AUDIT: Alcohol use disorders identification test
CASI: Computerized alcohol Screening brief Intervention, and referral to treatment
CDC: Centers for disease control and prevention
ED: Emergency department
e-SBI: Electronic screening and brief intervention
SBI: Screening and brief intervention
USPSTF: United States preventive services task force
